# Crystal structure and Hirshfeld surface analysis of 3,3′-[ethane-1,2-diylbis(­oxy)]bis­(5,5-di­methyl­cyclo­hex-2-en-1-one) including an unknown solvate

**DOI:** 10.1107/S2056989024004286

**Published:** 2024-05-17

**Authors:** Nurlana D. Sadikhova, Mehmet Akkurt, Valeh M. Ismayilov, Niftali N. Yusubov, Khudayar I. Hasanov, Ajaya Bhattarai

**Affiliations:** a Department of Chemistry, Baku State University, Z. Khalilov Str. 23, Az 1148 Baku, Azerbaijan; b Department of Physics, Faculty of Sciences, Erciyes University, 38039 Kayseri, Türkiye; c Western Caspian University, Istiqlaliyyat Street 31, AZ1001, Baku, Azerbaijan; d Azerbaijan Medical University, Scientific Research Centre (SRC), A. Kasumzade St. 14. AZ 1022, Baku, Azerbaijan; e Department of Chemistry, M.M.A.M.C (Tribhuvan University) Biratnagar, Nepal; Institute of Chemistry, Chinese Academy of Sciences

**Keywords:** crystal structure, β-diketones, dimers, weak inter­actions, Hirshfeld surface analysis

## Abstract

The title mol­ecule, C_18_H_26_O_4_, consists of two symmetrical halves related by the inversion centre at the mid-point of the central –C—C– bond. The hexene ring adopts an envelope conformation. In the crystal, the mol­ecules are connected into dimers by C—H⋯O hydrogen bonds with *R* ^2^_2_(8) ring motifs, forming zigzag ribbons along the *b*-axis direction. According to a Hirshfeld surface analysis, H⋯H (68.2%) and O⋯H/H⋯O (25.9%) inter­actions are the most significant contributors to the crystal packing. The contribution of some disordered solvent to the scattering was removed using the SQUEEZE routine [Spek (2015 ▸). *Acta Cryst*. C**71**, 9–18] in *PLATON*. The solvent contribution was not included in the reported mol­ecular weight and density.

## Abstract 2

In the crystal, the mol­ecules are connected into dimers by C—H⋯O hydrogen bonds with 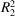 (8) ring motifs, forming zigzag ribbons along the *b*-axis direction.

## 1. Chemical context

β-Diketones have been employed as versatile synthetic precursors for the synthesis of new functional materials, such as catalysts, ionophores, heterocycles, organic conductors as well as pharmaceuticals (Abdelhamid *et al.*, 2011 ▸; Afkhami *et al.*, 2017 ▸; Khalilov *et al.*, 2021 ▸; Maharramov *et al.*, 2010 ▸; Martins *et al.*, 2017 ▸; Safavora *et al.*, 2019 ▸). For example, aryl­hydrazones of β-diketones have been widely used in coordination chemistry for a long time and have recently been the object of increasing attention as constituents of polydentate ligands in metallo-supra­molecular chemistry (Gurbanov *et al.*, 2018 ▸, 2020 ▸; Kopylovich *et al.*, 2012*a* ▸,*b* ▸; Mac Leod *et al.*, 2012 ▸; Mahmoudi *et al.*, 2017*a* ▸,*b* ▸, 2019 ▸). The reactivity of β-diketones as enols or ketones can also be used as a synthetic strategy to access new organic materials (Yamabe *et al.*, 2004 ▸). Moreover, bridging of two β-diketone moieties into one mol­ecule can improve their properties as well as the number of coordination and non-covalent sites (Shixaliyev *et al.*, 2019 ▸). 
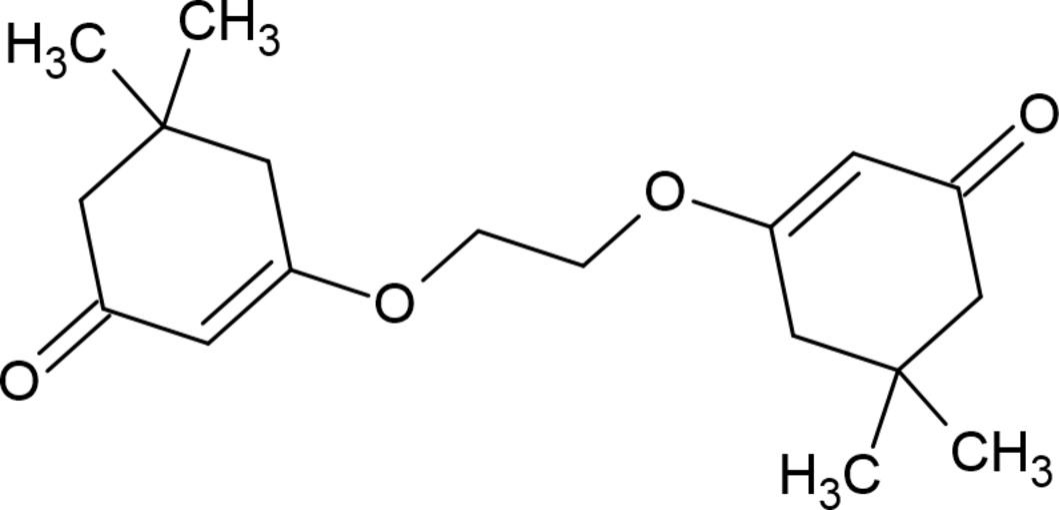


We have bridged two dimedone mol­ecules into 3,3′-[ethane-1,2-diylbis(­oxy)]bis­(5,5-di­methyl­cyclo­hex-2-en-1-one) *via* reaction with di­chloro­ethane, and undertaken a full characterization, including X-ray analysis.

## 2. Structural commentary

**Figure 1:**
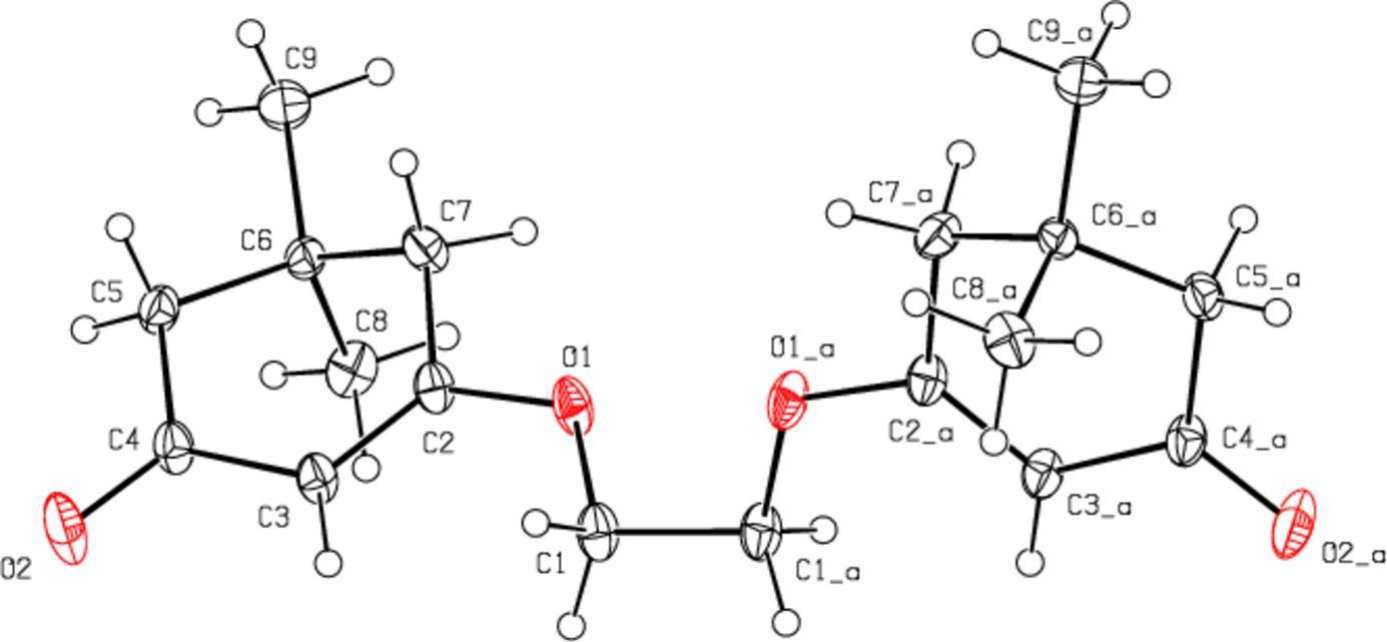
The mol­ecular structure of the title compound. Displacement ellipsoids are drawn at the 30% probability level.

The title compound (Fig. 1 ▸) consists of two symmetrical halves related by the inversion centre at the mid-point of the central –C—C– bond. The hexene ring (C2–C7) in the mol­ecule adopts an envelope conformation [the puckering parameters (Cremer & Pople, 1975 ▸) are *Q*_T_ = 0.4488 (15) Å, θ = 127.49 (19)°, φ = 60.6 (2)°]. The geometric parameters of the title compound are normal and comparable to those of the related compound listed in the *Database survey* section.

## 3. Supra­molecular features and Hirshfeld surface analysis

**Figure 2:**
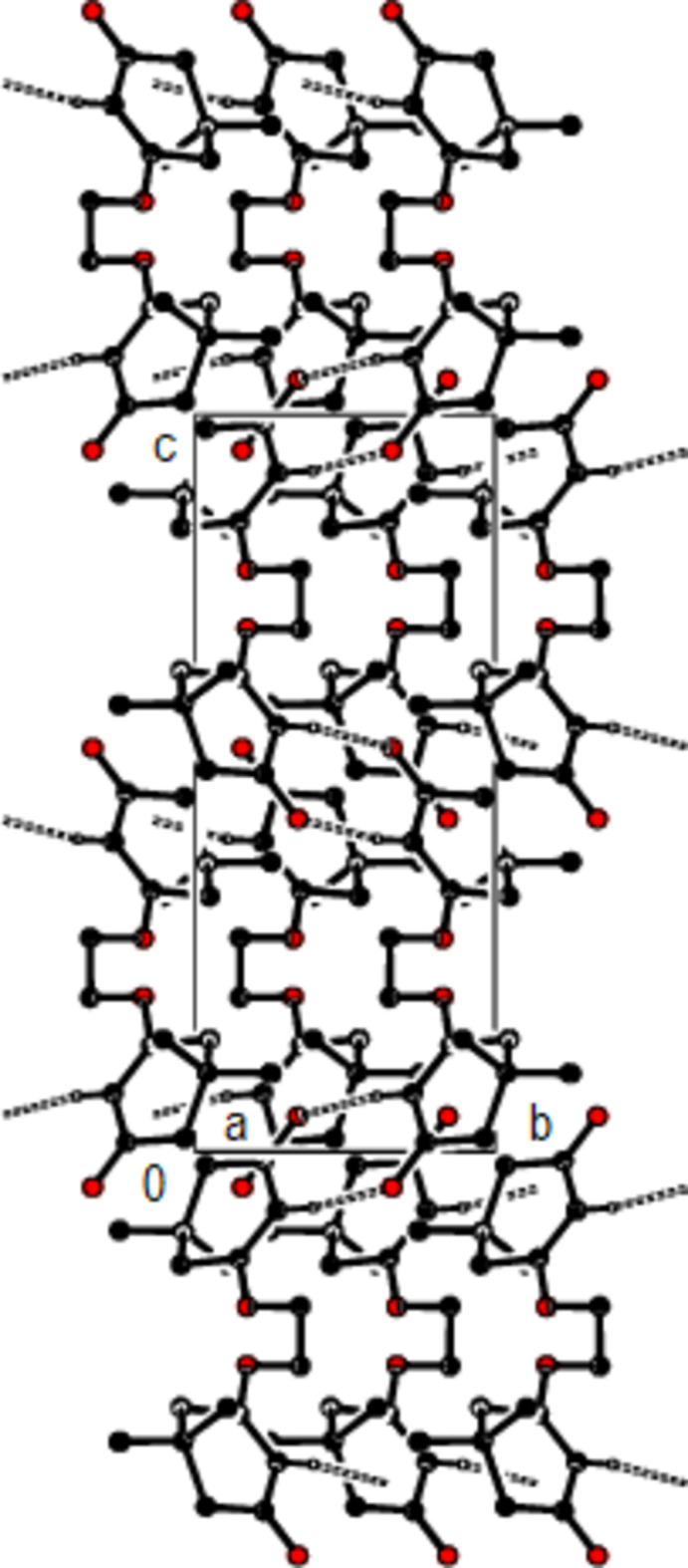
A partial view down the *a* axis of the C—H⋯O hydrogen bonds (dashed lines) in the title compound.

In the crystal, the mol­ecules are connected into dimers by C—H⋯O hydrogen bonds with 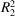 (8) ring motifs, forming zigzag ribbons along the *b*-axis direction (Bernstein *et al.*, 1995 ▸; Table 1 ▸; Figs. 2 ▸, 3 ▸ and 4 ▸). These ribbons are connected *via* van der Waals inter­actions, ensuring crystal cohesion.

**Figure 5:**
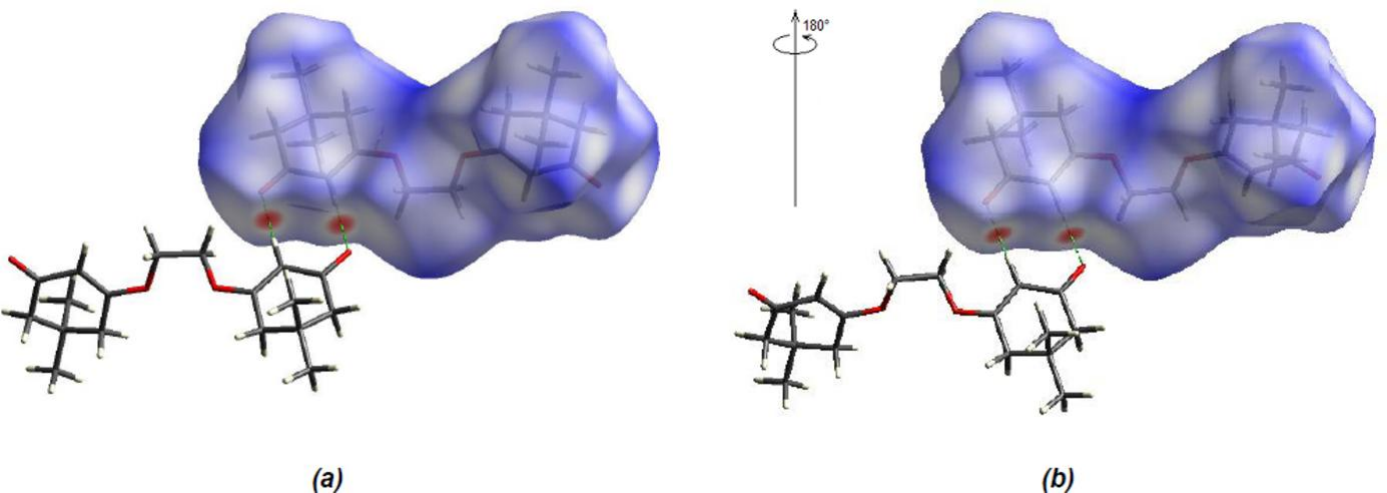
Front and back views of the three-dimensional Hirshfeld surfaces of the title compound.

**Figure 6:**
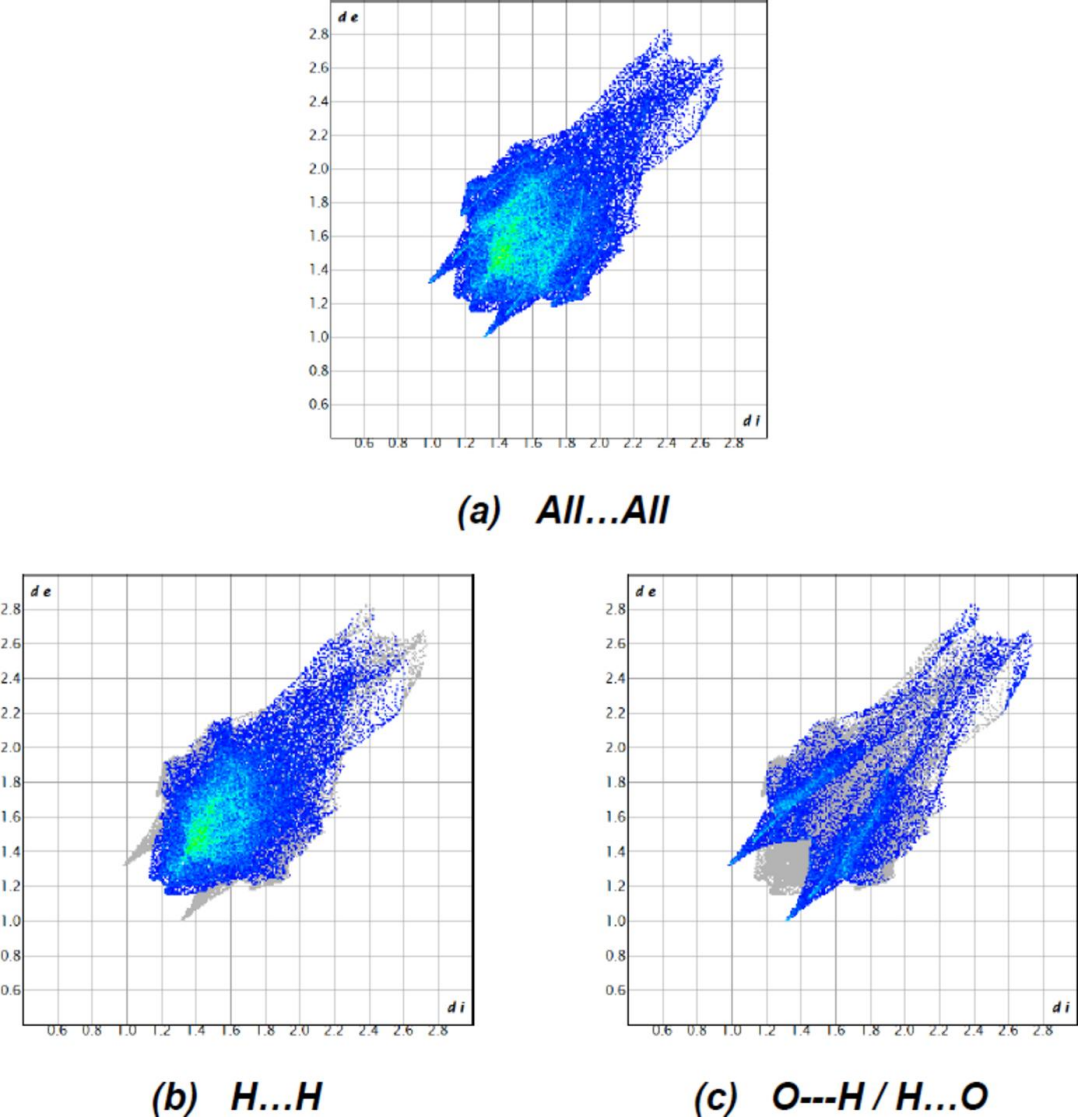
The full two-dimensional fingerprint plots for of the title compound, showing (*a*) all inter­actions, and delineated into (*b*) H⋯H and (*c*) O⋯H / H⋯O inter­actions. The *d*_i_ and *d*_e_ values are the closest inter­nal and external distances (in Å) from given points on the Hirshfeld surface.

In order to visualize and qu­antify the inter­molecular inter­actions, a Hirshfeld surface analysis was performed using *Crystal Explorer 17.5* (Spackman *et al.*, 2021 ▸), which was also used to generate the associated two-dimensional fingerprint plots. The Hirshfeld surfaces were mapped over *d*_norm_ in the range −0.2098 (red) to +1.6767 (blue) a.u. (Fig. 5 ▸). The most important inter­atomic contact is H⋯H as it makes the highest contribution to the crystal packing (68.2%, Fig. 6 ▸ *b*). The other major contributor is the O⋯H/H⋯O (25.9%, Fig. 6 ▸ *c*) inter­action. Other smaller contributions are made by C⋯H/H⋯C (5.5%) and O⋯O (0.4%) inter­actions.

## 4. Database survey

A search of the Cambridge Structural Database (CSD, Version 5.43, last update November 2022; Groom *et al.*, 2016 ▸) for the six-membered *cyclo­hexene* ring yielded nine compounds related to the title compound, *viz*. CSD refcodes WOMWUU (Naghiyev *et al.*, 2024 ▸), UPOMOE (Naghiyev *et al.*, 2021 ▸), ZOMDUD (Gein *et al.*, 2019 ▸), PEWJUZ (Fatahpour *et al.*, 2018 ▸), OZUKAX (Tkachenko *et al.*, 2014 ▸), IFUDOD (Gein *et al.*, 2007 ▸), IWEVOV (Mohan *et al.*, 2003 ▸), IWEVUB (Mohan *et al.*, 2003 ▸) and HALROB (Ravikumar & Mehdi, 1993 ▸).

WOMWUU, UPOMOE and ZOMDUD crystallize in the monoclinic space group *P*2_1_/*c*, with *Z* = 4, PEWJUZ in *I*2/*c* with *Z* = 4, IFUDOD, HALROB and IWEVUB in *P*2_1_/*n* with *Z* = 4, and IWEVOV and OZUKAX in the ortho­rhom­bic space group *Pbca* with *Z* = 8. In WOMWUU, mol­ecules are connected by inter­molecular C—H⋯S hydrogen bonds with 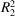 (10) ring motifs, forming ribbons along the *b*-axis direction. C—H⋯π inter­actions consolidate the ribbon structure while van der Waals forces between the ribbons ensure the cohesion of the crystal structure. In UPOMOE, the central cyclo­hexane ring adopts a chair conformation. In the crystal, mol­ecules are linked by N—H⋯O, C—H⋯O and C—H⋯N hydrogen bonds, forming mol­ecular layers parallel to the *bc* plane, which are connected by van der Waals inter­actions between them. In ZOMDUD, mol­ecules are linked by inter­molecular N—H⋯O and C—H⋯O hydrogen bonds, forming a three-dimensional network. C—H⋯π inter­actions are also observed. In PEWJUZ, mol­ecules are linked by inter­molecular N—H⋯O and C—H⋯O hydrogen bonds, forming sheets parallel to the *bc* plane. C—H⋯π inter­actions are also observed. In OZUKAX, mol­ecules are linked by inter­molecular N—H⋯O and C—H⋯O hydrogen bonds, forming sheets parallel to the *ac* plane. C—H⋯π inter­actions are also observed. Inter­molecular O—H⋯O hydrogen bonds consol­idate the crystal structure. There are no classical hydrogen bonds in the crystal of IFUDOD where inter­molecular C—H⋯O contacts and weak C—H⋯π inter­actions lead to the formation of a three-dimensional network. In the crystal of IWEVOV, the mol­ecules pack such that both carbonyl O atoms participate in hydrogen-bond formation with symmetry-related amide nitro­gen atoms present in the carbamoyl substituents, forming N—H⋯O hydrogen bonds in a helical arrangement. In the crystal, the phenyl rings are positioned so as to favour edge-to-edge aromatic stacking. When the crystal packing is viewed normal to the *ac* plane, it reveals a ‘wire-mesh’ type hydrogen-bond network. In the crystal of IWEVUB, unlike in IWEVOV where both carbonyl O atoms participate in hydrogen bonding, only one of the carbonyl oxygen atoms participates in inter­molecular N—H⋯O hydrogen bonding while the other carbonyl oxygen participates in a weak C—H⋯O inter­action. In addition, one of the amide nitro­gen atoms participates in N—H⋯O hydrogen bonding with the hydroxyl oxygen atom, linking the mol­ecules in a helical arrangement, which is similar to that in the structure of IWEVOV. As observed in the structure of IWEVOV, the packing of the mol­ecules viewed normal to the *ab* plane resembles a ‘wire-mesh’ arrangement of the mol­ecules. In the crystal of HALROB, the amide carbonyl groups are oriented in different directions with respect to the cyclo­hexa­none ring. These orientations of the carboxamide groups facilitate the formation of an intra­molecular O—H⋯O hydrogen bond. The mol­ecules are packed such that chains are formed along the *b*-axis direction. These chains are held together by N—H⋯O hydrogen bonds.

## 5. Synthesis and crystallization

0.12 mol of di­chloro­ethane were added drop by drop to a mixture of 0.12 mol of dimedone and 0.25 mol of K_2_CO_3_ in 50 mL of DMSO. The reaction mixture was held for 12 h at 353 K then cooled to room temperature, water added and extracted with ethyl ether. The extract was dried with MgSO_4_, the solvent was distilled off, and the residue was distilled under vacuum. Crystals suitable for X-ray analysis were obtained by evaporation of a di­methyl­formamide solution. Colourless solid (65%); m.p. 416–418 K. Analysis calculated for C_18_H_26_O_4_ (*M* = 306.40): C 70.56, H 8.55; found: C 70.52, H 8.49%. ^1^H NMR (300 MHz, DMSO-*d*_6_) δ 0.99 (12H, 4CH_3_), 2.12 and 2.30 (8H, 4CH_2_), 4.16 (4H, 2CH_2_) and 5.36 (2H, 2CH). ^13^C NMR (75 MHz, DMSO-*d*_6_) δ 27.72 (4CH_3_), 32.12 (2C_*ipso*_), 41.78 (2CH_2_), 50.25 (2CH_2_), 66.37 (2CH_2_), 101.44 (2CH), 175.18 (2C—O) and 197.89 (2C=O).

## 6. Refinement

Crystal data, data collection and structure refinement details are summarized in Table 2 ▸. All H atoms were placed in calculated positions (C—H = 0.95–0.99 Å) and allowed to ride on their carrier atoms, with *U*_iso_= 1.2 or 1.5*U*_eq_(C). The residual electron density was difficult to model and therefore the SQUEEZE routine (Spek, 2015 ▸) in *PLATON* (Spek, 2020 ▸) was used to remove the contribution of the electron density in the solvent region from the intensity data and the solvent-free model was employed for the final refinement. The solvent formula mass and unit-cell characteristics were not taken into account during refinement. The cavity of volume *ca* 77 Å^3^ (*ca* 4.4% of the unit-cell volume) contains approximately 11 electrons. A suitable solvent with this electron number may be about four dimethylformamide molecules per unit cell.

## Supplementary Material

Crystal structure: contains datablock(s) I. DOI: 10.1107/S2056989024004286/nx2010sup1.cif

Structure factors: contains datablock(s) I. DOI: 10.1107/S2056989024004286/nx2010Isup2.hkl

Supporting information file. DOI: 10.1107/S2056989024004286/nx2010Isup3.cml

CCDC reference: 2354123

Additional supporting information:  crystallographic information; 3D view; checkCIF report

## Figures and Tables

**Figure 3:**
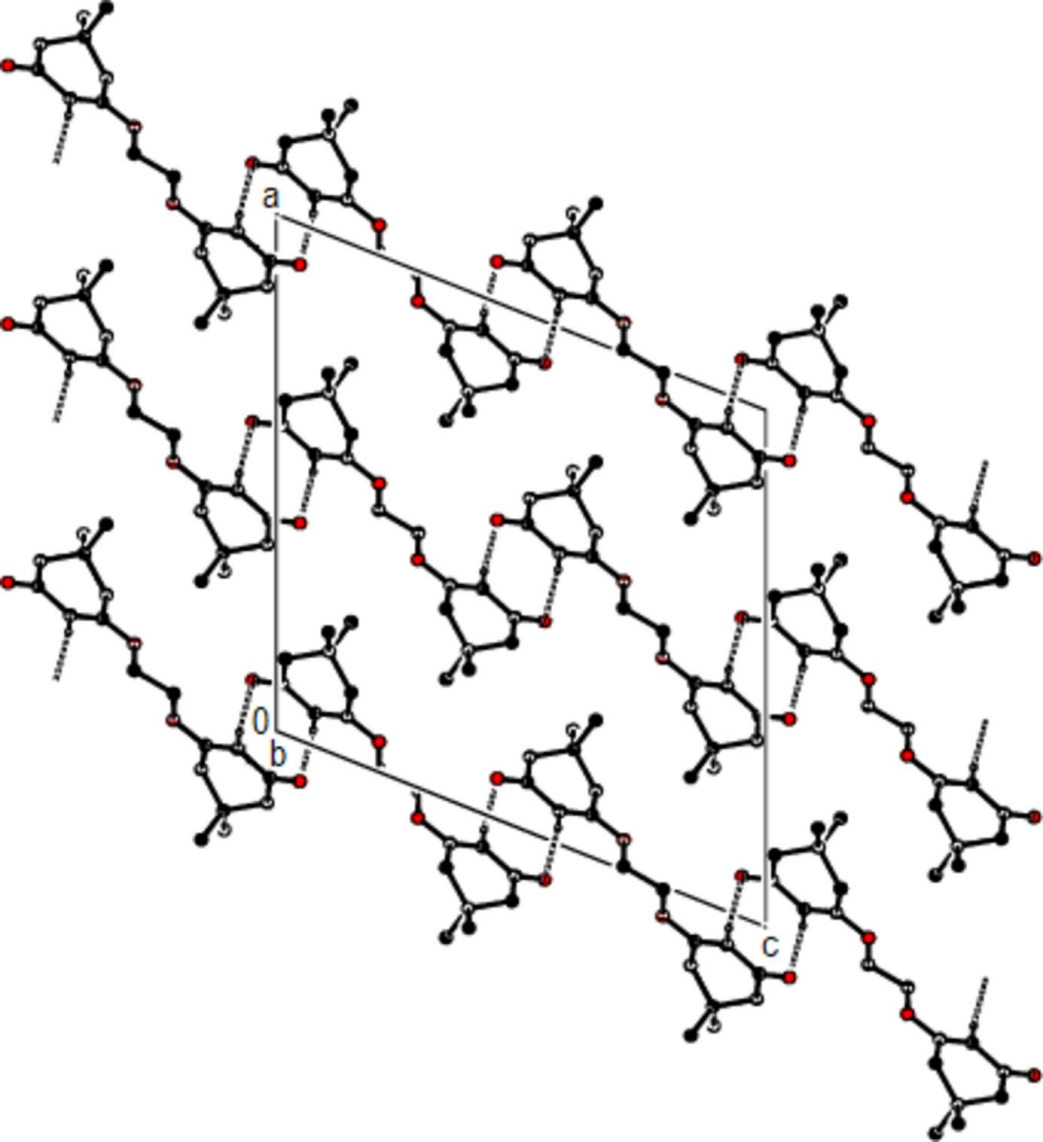
Partial packing of the title compound, viewed down the *b* axis, showing C—H⋯O hydrogen-bonded inversion-dimers with *R* ^2^_2_(8) graph-set motifs; H-atoms not involved in hydrogen bonds have been excluded for clarity.

**Figure 4:**
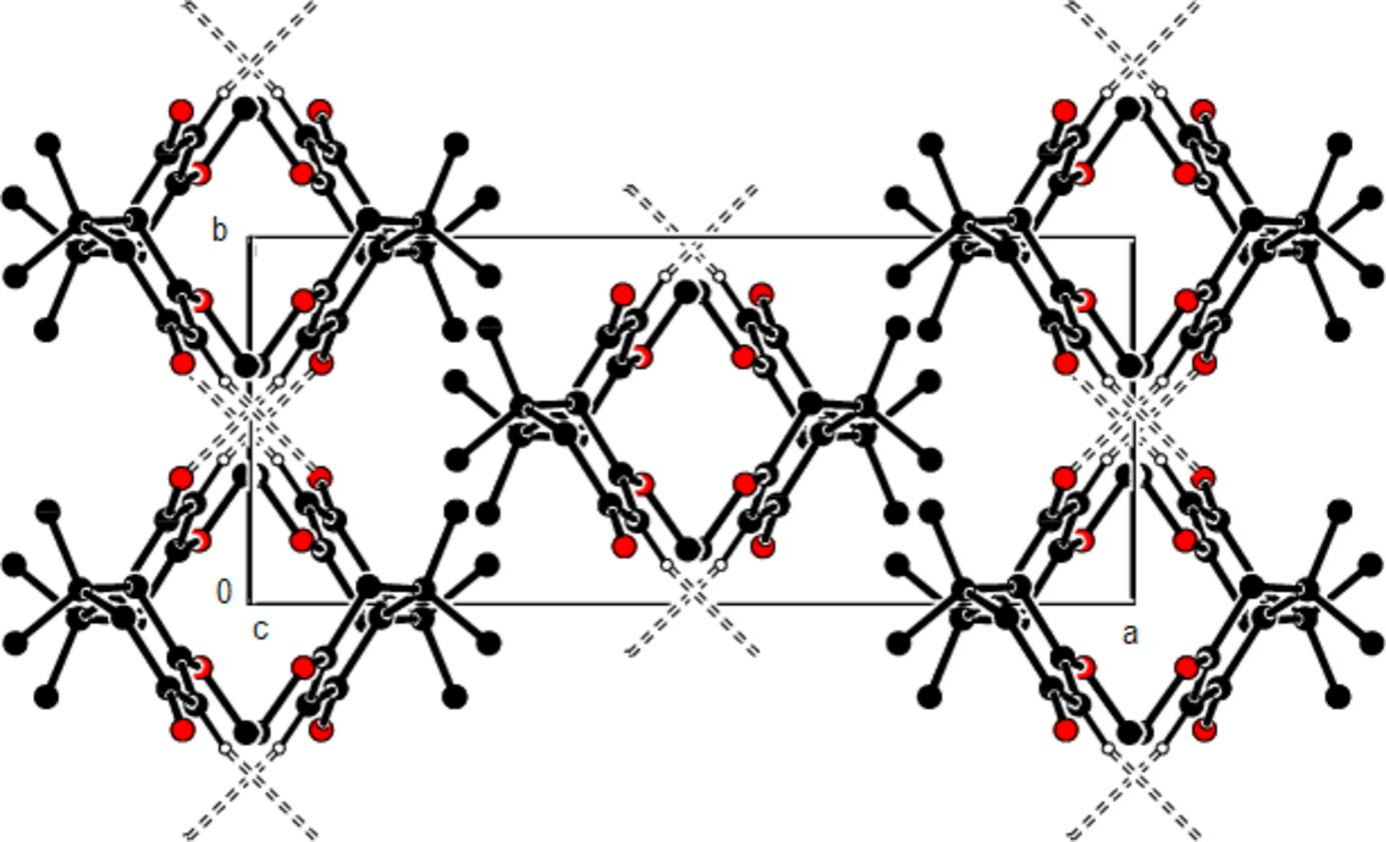
A partial view down the *c* axis of the C—H⋯O hydrogen bonds (dashed lines) in the title compound.

**Table 1:** Hydrogen-bond geometry (Å, °)

*D*—H⋯*A*	*D*—H	H⋯*A*	*D*⋯*A*	*D*—H⋯*A*
C3—H3⋯O2^i^	0.95	2.46	3.391 (2)	168

Symmetry code: (i) 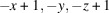 .

**Table 2:** Experimental details

Crystal data	
Chemical formula	C_18_H_26_O_4_
*M*_r_	306.39
Crystal system, space group	Monoclinic, *C*2/*c*
Temperature (K)	150
*a*, *b*, *c* (Å)	16.9184 (13), 6.5230 (5), 17.2645 (11)
β (°)	111.822 (4)
*V* (Å^3^)	1768.8 (2)
*Z*	4
Radiation type	Mo *K*α
μ (mm^−1^)	0.08
Crystal size (mm)	0.33 × 0.29 × 0.18

Data collection	
Diffractometer	Bruker APEXII CCD
Absorption correction	Multi-scan (*SADABS*; Krause *et al.*, 2015 ▸)
*T*_min_, *T*_max_	0.966, 0.980
No. of measured, independent and observed [*I* > 2σ(*I*)] reflections	10346, 2111, 1559
*R*_int_	0.046
(sin θ/λ)_max_ (Å^−1^)	0.659

Refinement	
*R*[*F* ^2^ > 2σ(*F* ^2^)], *wR*(*F* ^2^), *S*	0.045, 0.118, 1.04
No. of reflections	2111
No. of parameters	102
H-atom treatment	H-atom parameters constrained
Δρ_max_, Δρ_min_ (e Å^−3^)	0.26, −0.20

Computer programs: *APEX4* and *SAINT* (Bruker, 2018 ▸), *SHELXT* (Sheldrick, 2015*a* ▸), *SHELXL2018* (Sheldrick, 2015*b* ▸), *ORTEP-3 for Windows* (Farrugia, 2012 ▸) and *PLATON* (Spek, 2020 ▸).

## supplementary crystallographic information

### Crystal data

**Table:**

C_18_H_26_O_4_	*F*(000) = 664
*M**_r_* = 306.39	*D*_x_ = 1.151 Mg m^−^^3^
Monoclinic, *C*2/*c*	Mo *K*α radiation, λ = 0.71073 Å
*a* = 16.9184 (13) Å	Cell parameters from 2235 reflections
*b* = 6.5230 (5) Å	θ = 2.5–27.7°
*c* = 17.2645 (11) Å	µ = 0.08 mm^−^^1^
β = 111.822 (4)°	*T* = 150 K
*V* = 1768.8 (2) Å^3^	Prism, colourless
*Z* = 4	0.33 × 0.29 × 0.18 mm

### Data collection

**Table:**

Bruker APEXII CCD diffractometer	1559 reflections with *I* > 2σ(*I*)
φ and ω scans	*R*_int_ = 0.046
Absorption correction: multi-scan (SADABS; Krause *et al.*, 2015)	θ_max_ = 27.9°, θ_min_ = 2.5°
*T*_min_ = 0.966, *T*_max_ = 0.980	*h* = −22→22
10346 measured reflections	*k* = −8→8
2111 independent reflections	*l* = −22→22

### Refinement

**Table:**

Refinement on *F*^2^	Primary atom site location: structure-invariant direct methods
Least-squares matrix: full	Secondary atom site location: difference Fourier map
*R*[*F*^2^ > 2σ(*F*^2^)] = 0.045	Hydrogen site location: inferred from neighbouring sites
*wR*(*F*^2^) = 0.118	H-atom parameters constrained
*S* = 1.04	*w* = 1/[σ^2^(*F*_o_^2^) + (0.0456*P*)^2^ + 1.1303*P*] where *P* = (*F*_o_^2^ + 2*F*_c_^2^)/3
2111 reflections	(Δ/σ)_max_ < 0.001
102 parameters	Δρ_max_ = 0.26 e Å^−^^3^
0 restraints	Δρ_min_ = −0.20 e Å^−^^3^

### Special details

**Table:**

Geometry. All esds (except the esd in the dihedral angle between two l.s. planes) are estimated using the full covariance matrix. The cell esds are taken into account individually in the estimation of esds in distances, angles and torsion angles; correlations between esds in cell parameters are only used when they are defined by crystal symmetry. An approximate (isotropic) treatment of cell esds is used for estimating esds involving l.s. planes.

### Fractional atomic coordinates and isotropic or equivalent isotropic displacement parameters (Å^2^)

**Table:**

	*x*	*y*	*z*	*U*_iso_*/*U*_eq_	
O1	0.44150 (6)	0.32631 (17)	0.28986 (6)	0.0333 (3)	
O2	0.42054 (9)	0.1572 (2)	0.54805 (7)	0.0545 (4)	
C6	0.30515 (9)	0.5396 (2)	0.39219 (8)	0.0252 (3)	
C2	0.41732 (8)	0.3534 (2)	0.35542 (8)	0.0264 (3)	
C3	0.43558 (8)	0.2261 (2)	0.42109 (8)	0.0276 (3)	
H3	0.468878	0.106836	0.424213	0.033*	
C7	0.36773 (9)	0.5477 (2)	0.34683 (9)	0.0311 (3)	
H7A	0.335662	0.575102	0.286885	0.037*	
H7B	0.407869	0.662711	0.369541	0.037*	
C5	0.35360 (9)	0.4640 (2)	0.48148 (8)	0.0296 (3)	
H5A	0.392750	0.573764	0.513085	0.036*	
H5B	0.312185	0.439501	0.508610	0.036*	
C4	0.40447 (9)	0.2706 (2)	0.48729 (8)	0.0299 (3)	
C1	0.49293 (9)	0.1495 (3)	0.29054 (9)	0.0316 (4)	
H1A	0.463346	0.022376	0.295832	0.038*	
H1B	0.548105	0.157036	0.338214	0.038*	
C8	0.23173 (9)	0.3930 (3)	0.34703 (10)	0.0350 (4)	
H8A	0.201336	0.441546	0.289903	0.052*	
H8B	0.192439	0.388104	0.376769	0.052*	
H8C	0.254452	0.255480	0.345518	0.052*	
C9	0.26825 (12)	0.7527 (3)	0.39343 (10)	0.0429 (4)	
H9A	0.314317	0.847356	0.423877	0.064*	
H9B	0.227197	0.745890	0.421145	0.064*	
H9C	0.239567	0.801562	0.336078	0.064*	

### Atomic displacement parameters (Å^2^)

**Table:**

	*U*^11^	*U*^22^	*U*^33^	*U*^12^	*U*^13^	*U*^23^
O1	0.0318 (5)	0.0481 (7)	0.0280 (5)	0.0114 (5)	0.0202 (4)	0.0110 (5)
O2	0.0716 (9)	0.0690 (9)	0.0366 (6)	0.0442 (7)	0.0362 (6)	0.0280 (6)
C6	0.0289 (7)	0.0247 (7)	0.0238 (7)	0.0061 (6)	0.0121 (6)	0.0007 (5)
C2	0.0206 (6)	0.0374 (8)	0.0251 (7)	0.0023 (6)	0.0130 (5)	0.0031 (6)
C3	0.0255 (7)	0.0352 (8)	0.0259 (7)	0.0101 (6)	0.0141 (6)	0.0058 (6)
C7	0.0341 (8)	0.0309 (8)	0.0319 (7)	0.0035 (6)	0.0163 (6)	0.0082 (6)
C5	0.0342 (8)	0.0323 (8)	0.0244 (7)	0.0065 (6)	0.0134 (6)	−0.0016 (6)
C4	0.0291 (7)	0.0393 (8)	0.0237 (7)	0.0107 (6)	0.0124 (6)	0.0056 (6)
C1	0.0259 (7)	0.0468 (9)	0.0274 (7)	0.0067 (6)	0.0160 (6)	0.0036 (6)
C8	0.0247 (7)	0.0441 (9)	0.0355 (8)	0.0049 (6)	0.0106 (6)	−0.0037 (7)
C9	0.0584 (11)	0.0332 (9)	0.0406 (9)	0.0173 (8)	0.0223 (8)	0.0045 (7)

### Geometric parameters (Å, º)

**Table:**

O1—C2	1.3507 (16)	C5—C4	1.5096 (19)
O1—C1	1.4423 (17)	C5—H5A	0.9900
O2—C4	1.2284 (17)	C5—H5B	0.9900
C6—C9	1.527 (2)	C1—C1^i^	1.505 (3)
C6—C8	1.532 (2)	C1—H1A	0.9900
C6—C5	1.5336 (19)	C1—H1B	0.9900
C6—C7	1.5340 (19)	C8—H8A	0.9800
C2—C3	1.3454 (19)	C8—H8B	0.9800
C2—C7	1.496 (2)	C8—H8C	0.9800
C3—C4	1.4543 (18)	C9—H9A	0.9800
C3—H3	0.9500	C9—H9B	0.9800
C7—H7A	0.9900	C9—H9C	0.9800
C7—H7B	0.9900		
			
C2—O1—C1	117.88 (10)	C6—C5—H5B	108.6
C9—C6—C8	108.50 (12)	H5A—C5—H5B	107.6
C9—C6—C5	110.31 (11)	O2—C4—C3	121.41 (13)
C8—C6—C5	109.89 (12)	O2—C4—C5	119.97 (12)
C9—C6—C7	109.84 (12)	C3—C4—C5	118.59 (12)
C8—C6—C7	110.15 (11)	O1—C1—C1^i^	107.24 (10)
C5—C6—C7	108.15 (11)	O1—C1—H1A	110.3
C3—C2—O1	125.39 (13)	C1^i^—C1—H1A	110.3
C3—C2—C7	123.44 (12)	O1—C1—H1B	110.3
O1—C2—C7	111.17 (11)	C1^i^—C1—H1B	110.3
C2—C3—C4	120.14 (13)	H1A—C1—H1B	108.5
C2—C3—H3	119.9	C6—C8—H8A	109.5
C4—C3—H3	119.9	C6—C8—H8B	109.5
C2—C7—C6	112.86 (11)	H8A—C8—H8B	109.5
C2—C7—H7A	109.0	C6—C8—H8C	109.5
C6—C7—H7A	109.0	H8A—C8—H8C	109.5
C2—C7—H7B	109.0	H8B—C8—H8C	109.5
C6—C7—H7B	109.0	C6—C9—H9A	109.5
H7A—C7—H7B	107.8	C6—C9—H9B	109.5
C4—C5—C6	114.46 (11)	H9A—C9—H9B	109.5
C4—C5—H5A	108.6	C6—C9—H9C	109.5
C6—C5—H5A	108.6	H9A—C9—H9C	109.5
C4—C5—H5B	108.6	H9B—C9—H9C	109.5
			
C1—O1—C2—C3	−1.6 (2)	C9—C6—C5—C4	−170.57 (13)
C1—O1—C2—C7	177.57 (12)	C8—C6—C5—C4	69.86 (16)
O1—C2—C3—C4	−178.91 (13)	C7—C6—C5—C4	−50.42 (16)
C7—C2—C3—C4	2.0 (2)	C2—C3—C4—O2	−179.70 (15)
C3—C2—C7—C6	−28.0 (2)	C2—C3—C4—C5	−1.5 (2)
O1—C2—C7—C6	152.85 (12)	C6—C5—C4—O2	−154.38 (15)
C9—C6—C7—C2	170.39 (13)	C6—C5—C4—C3	27.4 (2)
C8—C6—C7—C2	−70.16 (15)	C2—O1—C1—C1^i^	177.15 (12)
C5—C6—C7—C2	49.95 (16)		

Symmetry code: (i) −*x*+1, *y*, −*z*+1/2.

### Hydrogen-bond geometry (Å, º)

**Table:**

*D*—H···*A*	*D*—H	H···*A*	*D*···*A*	*D*—H···*A*
C3—H3···O2^ii^	0.95	2.46	3.391 (2)	168

Symmetry code: (ii) −*x*+1, −*y*, −*z*+1.
